# *‘People ring because they’re frightened’:* findings from a realist evaluation on the impact of timely responsive care at home at the end of life

**DOI:** 10.1186/s12904-025-01826-y

**Published:** 2025-07-14

**Authors:** Kathryn McEwan, Joanne Atkinson, Amanda Clarke, Angela Bate, Caroline Jeffery, Sonia Dalkin

**Affiliations:** University of Northumbria, Newcastle upon Tyne, England

**Keywords:** Palliative care, End of life, Rapid response services, Realist evaluation, Transitions theory, Home death, Compassion, Community, Caregivers

## Abstract

**Background:**

Rapid response services (RRS) support patients who wish to die at home, providing flexible, timely, and specialist care. These services are regionally variable yet are valued by patients and caregivers in often uncertain end-of-life situations. Research on their effectiveness and implementation to date is limited. This study explores how RRS are experienced in practice and identifies key contexts and mechanisms underpinning their impact.

**Objectives:**

This study aimed to understand how different service models of RRS function, who they work for, how and why. By exploring patient, caregiver, and staff perspectives, we sought to refine programme theories and provide evidence-based recommendations for service improvement and policy development.

**Design:**

A realist evaluation approach was used to examine how, why, and in what contexts RRS provide end-of-life care. Through iterative theory development and refinement, we identified key generative mechanisms and the contexts in which they trigger outcomes.

**Methods:**

Qualitative data were collected via realist theory driven semi-structured focus groups and interviews with 36 participants across two sites, each operating a distinct RRS model. Data were analysed using a retroductive context-mechanism-outcome (CMO) framework and informed by Transitions Theory.

**Results:**

Six programme theories were developed, highlighting the central role of continuity of care in enabling positive end-of-life experiences. A sense of ‘being known’ by RRS staff facilitated smooth transitions, reduced distress, and fostered trust. Timely, responsive care, particularly at night, was valued, whereas gatekeeping, fragmented service models, and inequities in access (especially for non-cancer patients) created barriers.

**Conclusions:**

Patients and caregivers valued holistic, relationship-centred care that provided emotional security alongside practical support. However, service inconsistencies, late transitions into palliative care, and systemic inequities limit accessibility. Findings highlight the need for early engagement, integrated service models, and 24/7 specialist care, ensuring greater continuity and equity in home-based end-of-life care.

**Supplementary Information:**

The online version contains supplementary material available at 10.1186/s12904-025-01826-y.

## Introduction

Rapid response services (RRS) are an example of community end-of-life care support made available to those who choose to die at home. They provide care and support to patients and their caregivers in circumstances where death has become increasingly medicalised, removed from families and communities and into health systems [1]. RRS are typically delivered by specialist palliative care teams, community nurses, or collaborative teams which are embedded in acute, community, or primary care settings, depending on local service configuration.

The preference for death at home is well documented. One recent survey found that 56% of respondents, in the last years of life, stated a preference to die at home, yet only 23% of deaths occurred at home [2]. A significant proportion of deaths continue to occur in hospitals. In England and Wales in 2023, up to 50.8% of deaths occurred in hospital, while up to 33.6% occurred at home [3]. To meet stated preferences, and support choice and dignity at the end-of-life, community end-of-life care must be available and accessible. Previous research has identified the need for significant improvement in community end-of-life care [4–9].

Recent research highlights that community out-of-hours palliative care is often'a patchwork of services', emphasising the variability and inconsistency in care delivery during evenings and weekends. This fragmentation can lead to disparities in patient and caregiver experiences, with some receiving timely and comprehensive support, while others encounter delays and gaps in care [10]. A lack of specialist overnight services and inconsistent out-of-hours provision continues to create barriers to home-based care, increasing reliance on emergency services during crises [11]. These inconsistencies emphasise the need for new evidence which demonstrates how RRS operate.

Caregivers want to know what they can expect to happen when supporting end-of-life and who they should contact for help [12]. Informal caregivers, predominantly family members, provide up to 90% of the care to those at home at the end-of-life [13]. Caregivers have evolving needs during this period and can often find themselves'navigating the caregivers'abyss'[14]. During this time, they find themselves managing multiple roles, facing emotional, physical and logistical challenges, needing to mobilise various resources, all while preparing for the end-of-life transition [14].

There are many assumptions made in the ‘choice’ of death at home, these can include that home is available and safe [15]. These assumptions are amplified by potential uncertainty around prognosis, differing disease trajectories, and fear or distrust of palliative care, which can all act as barriers to a timely transition into palliative and end of life care and discussion, and therefore subsequent service access and engagement. Practitioners attending to death and dying at home are'professional guests', who must navigate and negotiate boundaries between their professional status and their need to create connection and personalise care [16].

We bring new evidence to the literature on the strengths and limitations of different RRSs, including the resources expected of informal caregivers and the barriers and facilitators to service access. The study reported here set out to better understand how RRS operate in context, who they do and do not work for, how, and why [17], so palliative and end of life care services can respond to the challenges posed by an ageing population that require care and compassion at the end of life. In doing so, our work also responds to recent calls for greater integration of social science perspectives in palliative care research, highlighting how dying is shaped by systems, relationships, and inequality [18].

## Methods

### Design

Realist evaluation is a theory-driven approach that seeks to understand how and why complex interventions work (or not) in specific contexts [17]. The process can be set out over three phases; at the outset,’theory development’ is undertaken, where initial programme theories (IPTs) are hypothesised of how, in certain contexts, particular mechanisms may lead to different outcomes. ‘Theory testing’ follows, in this phase data is collected and analysed to test the IPTs; consequently, the programme theories are revised iteratively. In the last phase, ‘theory refinement’ produces refined theories that reflect the observed realities [19, 20]. For more detail of our ‘theory development’ phase, see previous paper [21].

### Data Collection

The three phases of this study occurred between April 2021 and April 2024. In Phase 1, literature scoping activity and early information gathering with stakeholders led to the IPT development. Phase 2 and 3, undertaken concurrently, included semi structured realist theory driven focus groups and interviews. Interview guides for health and social care professionals and informal caregivers (see Supplementary Files 3 and 4) were designed to focus on and interrogate key contexts, mechanisms and outcomes identified in phase 1 [22]. These guides were designed to query the ways in which the RRS works within different contexts, with prompts to specifically identify triggering mechanisms that lead to outcomes [23].

### Sample

Two sites delivering a RRS were included in the study. Model A was a situated in an acute setting, operating RRS 7am–7 pm daily, primarily supporting patients already known to the specialist palliative care team; Model B was a standalone, community-based service operating 24/7, delivering care exclusively in the community.

Gatekeepers were RRS staff who had an existing clinical relationship with the patient or caregiver. During home visits, they provided participant information sheets and collected verbal permission to be contacted, using consent-to-contact forms. These details were then collated in the offices and shared securely with the research team once a week for follow-up. Table 1 below provides our final participation numbers but also outlines the difference between contact details shared from the sites and the recruitment to interview for caregivers and patients. We address the potential contributing factors to this in limitations below.

**Table 1:** Final qualitative data participation

Participant Group	Site	Permission to Contact Details Referred	Interviews Completed
**Caregivers**	Model A	8	1
	Model B	13	5
**Total Caregivers**		21	6
**Patients**	Model A	5	0
	Model B	2	1
**Total Patients**		7	1
**RRS Staff**	Model A	-	6
	Model B	-	14
**Total RRS Staff**		-	20
**External Staff**	Model A	-	6
	Model B	-	3
**Total External Staff**		-	9
**Overall Total**			**36 Participants**

### Sample characteristics

Samples were purposive, based on the IPTs developed in Phase 1 [24]. We interviewed a range of RRS staff from both sites, as well as supporting or adjunct staff (we call ‘external staff’) who worked alongside or had knowledge of the RRS. See Table 2 for staff sample characteristics. We also undertook interviews with caregivers, although more were successfully recruited on Model B site than Model A, see Table 3 for characteristics.

**Table 2:** Staff participant roles

Participant Group	Participant Role	Total
**RRS Staff Participants**		
Service Model A – RRS Staff (Interviews)	RRS Registered Nurses	4
	Consultant	1
	**Total**	**5**
Service Model B – RRS Staff (Focus Groups)	RRS Registered Nurses	5
	Senior Healthcare Assistants	2
	Service Manager	1
	**Total**	**8**
Service Model B – RRS Staff (Interviews)	RRS Registered Nurses	4
	Senior Healthcare Assistants	
	**Total**	**6**
	**RRS Staff Participants Total**	**19**
**External Staff Participants**		
External Staff – Model A (Interviews)	Commissioner	1
	General Practitioners	2
	Hospice Consultant	1
	Hospital Consultant	1
	Out of Hours Registered Nurse	1
	**Total**	**6**
External Staff – Model B (Interviews)	Nurse Manager	1
	General Practitioner	1
	General Practitioner Practice Co-ordinator	1
	**Total**	**3**
	**External Staff Participant Total**	**9**

**Table 3:** Caregiver characteristics

RRS Service	Participant Characteristics	Total
**Model A**	Spouse Caregiver	1
**Model B**	Spouse Caregiver	2
	Adult Child Caregiver	3
	**Total Caregiver Participants**	**6**

### Data Analysis

In line with realist evaluation, data were analysed iteratively throughout phase 2 and 3. All interviews and focus groups were transcribed verbatim and anonymised by the researcher (KM) or a professional transcription service under a confidentiality agreement. At this point, they were added to NVivo 12 Pro for data management. To make the data analysis manageable, IPTs were grouped into early concept areas (e.g., being ‘on the books’, including/excluding factors, trust). Initial coding included comparing and contrasting accounts across the dataset, utilising inductive and deductive reasoning to identify dyads and triads of context, mechanism and outcomes [25]. We used constant comparison across the data set as it emerged alongside retroduction [26] to continually test and refine the IPTs. This testing included seeking what supported and refuted theories, refining theories as the data set grew, and continually challenging the initial propositions [17].

The researcher (KM) managed the data in NVivo and transferred anonymised sections of transcripts and coding into MS Teams so the full research group, including PPI representatives, could meet to consider and discuss the analysis progress, providing input, checks, and balances. Early searching of substantive theory for explanatory purposes led to the introduction of Transitions Theory into the analysis process. Transitions Theory draws attention to the nature and properties of change in health and illness, including contextual influences and patterns of response, what supports or hinders adjustment during key transition points (such as entry into palliative care or the final days of life), and the implications for healthcare services [27–29].

In this study, the theory helped us move beyond viewing care episodes as isolated events, instead conceptualising patient and caregivers experiences as unfolding across a journey of interlinked emotional, relational, and service-related transitions. This supported our analysis of how RRS could offer integrated and continuous care in contrast to fragmented or disjointed episodes of support. A structured matrix was developed on MS Excel to map and test the substantive theory, Transitions Theory [27], across iterative stages of analysis (see Supplementary file 5).

The study results are reported in line with RAMESES reporting guidelines for realist evaluation [30] see Supplementary File 1 for checklist.

### Reflexive Note on Terminology

We acknowledge ongoing debate over terminology used to describe those providing unpaid care, particularly the critique that the term'informal caregiver'may unintentionally minimise the expertise and responsibility involved [31]. Applebaum’s position, that the word ‘informal’ undermines the value of caregiving, arises from both scholarly and lived experience, and is a powerful call to adopt alternatives such as'care partners'or'family caregivers.'Yet, we retain the term'informal caregiver'in this paper with intentionality. It remains widely used in health and social care policy and research, providing continuity and analytic clarity. Importantly, we use the term not as a neutral descriptor but as a contested category, recognising that many individuals providing care do not identify as'carers','family', or'partners'[32]. These identity complexities are particularly important when applying transitions theory to caregiver experience; as such, our use of'informal caregiver'is pragmatic and critical, aligning with our methodological and theoretical commitments.

## Results

The overall study produced 6 refined programme theories from the original 8 IPTs; see Supplementary File 2 for details on IPT to PT development, including revisions, into final refined PTs and Table 4 for an overview. We set out the data for PT1 Skilled Communication in a previous paper [33]. In this paper, we set out the subsequent 5; PT2 Service Access, which identifies the barriers to different RRS due to gatekeeping and how continuity of care is achieved through different service models. PT3 Timely Response, which identifies the impact of the speed and nature of the response to crisis. PT4 12v24hr, how services support patients and caregivers through the challenge of the night. PT5 Beyond Cancer Care, the impact on RRS access of differing diagnosis and prognosis. PT6 Responsive Skilled Support, the implicit and explicit expectations RRS appear to have for caregivers and patients to allow for access.

**Table 4:** IPT-PT overview

IPT Title	PT Title
**IPT1** Communications	**PT1** Skilled Communication
**IPT2** Values	Ended – data contributed towards refinement of PT6 Responsive Skilled Support and PT5 Beyond Cancer Care
**IPT3** Access	**PT2** Service Access
**IPT4** Diverse Needs	**PT5** Beyond Cancer Care
**IPT5** Geography and Community	Ended – insufficient data to test the theory
**IPT6** 12v24hr	**PT4** 12v24hr
**IPT7** Timeliness	**PT3** Timely Response
**IPT8** Who	**PT6** Responsive Skilled Support

Each PT has an identified context, mechanism-resource, mechanism-response, and outcome which identifies the generative causation in action. Differing contexts, mechanisms, and outcomes are also labelled as such within the quotes below. At the end of each sub-section, the refined programme theory is stated in full in Tables 5, 6, 7, 8, 9.

**Table 5:** PT2 Service Access

PT2 Service access
Potential services users require straightforward and clear access to specialist palliative care at the end of life in the community, this could be through integrated or adjunct services (context), by providing proactive information sharing within and between acute and primary care (e.g. shared information systems or proactive action by staff to share information) (resource) rapid response services can overcome gatekeeping barriers (response) and improve potential to service access (outcome).
PT2.1 Gatekeeping and Professional Trust – Model A
Model A offers an integrated recognised specialist palliative care service in the community at end of life (e.g., integration with the acute sector and primary care, including the DN service who are the first port of call) (context) which allows for formal and informal information sharing and working practices (e.g., co-location with acute sector and DN’s, shared SystmOne, and attendance at MDT where necessary) (resource). Shared information systems support recognition of the boundaries, roles, and speciality of the service increasingpartners awareness, confidence, and professional trust (response) who, reassured, will be more likely to refer increasing opportunity to access the service (outcome).
PT2.2 Gatekeeping and Professional Trust – Model B
Model B offers an adjunct recognised specialist palliative care service in the community at end of life (e.g., offered alongside the DN service, who are the first port of call) (context) which allows for formal but restricted information sharing (e.g., facilitated through shared SystmOne access, outreach activity to engage with primary care, and attendance at MDT where invited) (resource). Proactive action by staff supports recognition of the boundaries, roles, and speciality of the service increasing partners awareness, confidence, and professional trust (response) and so, reassured, will be more likely to refer increasing opportunity to access the service (outcome).
PT2.3 Continuity - Model A
Model A provides a person-centred approach (e.g., focus is on the patient’s needs and wellbeing and interactions with other services but not as focused on caregivers) (context). Where services are aware that patients have significant palliative care needs, they have access to a planned palliative care service and an adjunct rapid response service that can respond to the urgent and out of hours needs of the patient (context), This service provides valued specialist care and works to maintain relationships between different teams and organisations to provide continuity of care (resource) until the patient dies. This develops relationships and supports caregivers to provide home care (reasoning), with positive impacts on patient and caregiver well-being (outcome) but doesn’t provide support for death and beyond for the carer (outcome).
PT2.4 Continuity – Model B
Model B provides a relationship centred care approach (e.g., incorporating the needs and well-being of not only the patient but also of their significant others, and prioritising relationships between professional staff) (context). It provides specialist and holistic care underpinned by a palliative ethos, which leads to a robust continuity of care to the patient and caregiver, throughout the patient and carer journey (including at death, post-death, and bereavement) (resource). This will lead to relationships of trust and care (reasoning) with positive impacts on patient and caregiver well-being and patient dignity (outcome).

**Table 6:** PT3 Timely Response

Death has become hidden from society and medicalised, but home deaths are the preference of many individuals; this means patients and caregivers may find dying at home emotionally daunting and practically challenging (context). Where there is a timely response guaranteed from a specialist palliative care service (one hour) with a home visit with no time deadline (i.e., not a 15/30 min timed stay) if required, and/or a telephone response for support for difficult or crisis situations (symptom management, distress, discomfort) (resource), provides emotional and practical reassurance, leading to improved confidence to patients and caregivers (response). Thisleads to less (attempts) at use of other generalist or emergency services (outcome) and/or improves perceived wellbeing for patients and caregivers (outcome).

**Table 7:** PT4 12v24hr

PT4.1–24hr Specialist Support: Patients approaching and caregivers caring for someone at the end of life, often find the night to be a worrying time due to a lack of service access (context), where patients and informal caregivers have access to 24hr care 7 days a week from the specialist palliative care team who can be contacted through 1 phone number and attend in person if required (resource), building relationships, they are reassured and confident to manage dying at home (response), improving perceived well-being (outcome). This reduces the likelihood of crisis situations and calls and ‘inappropriate’ hospital admissions (outcome).
PT4.2–12hr Planned Specialist Support: Patients approaching and caregivers caring for someone at the end of life, often find the night to be a worrying time due to a lack of service access (context), where a planned community specialist palliative service exists, with crisis responses during the day (12hr care 7 days a week), strategies can be developed with patients/caregivers to plan and prepare for overnight, with support from OOH DN/GP where necessary, who have shared information systems (resource). This builds relationships, and so patients and caregivers feeling reassured and confident to manage dying at home (response), improving perceived well-being (outcome). This reduces the likelihood of crisis situations and calls and ‘inappropriate’ hospital admissions (outcome).

**Table 8:** PT5 Beyond Cancer Care

Uncertainty around prognosis for non-malignant disease, as well as general fear and misunderstanding around palliation and end of life care (and their differences) in the wider population, can lead to a lack of awareness of, and timely transition into, palliative and end of life care (context). Well-funded, accessible services, rooted in primary care rather than aligned to the acute sector (resource), increase awareness, and improve access (response), leading to improved care for all patients and caregivers (outcome) and reduced hospital admissions (outcome).

**Table 9:** PT6 Responsive Skilled Support

Supporting death and dying at home, necessitates informal caregivers to have access to several resources (economic, physical, social, cultural, and emotional) which differ in each individual circumstance (context). Where specialist end of life care services, with prescribing capabilities, cultural competences and inclusive practices, are available to respond quickly and flexibly, without time limits, to all these potential support needs (resource), caregivers and services can ‘work together’ (resource), and patients and caregivers feel reassured, confident, and capable to continue to provide care at home, or to change their minds/course and access in-patient or Hospice care if appropriate (response). This leads to improved perceived well-being of patients and caregivers (outcome) and improved opportunities for death at home (outcome)	

Both Rapid Response Services (Model A and Model B) deliver care at home at the end of life, although their service model is different. The findings reflect these differences, sometimes providing a refined PT that is applicable to both services, or by instead providing two which reflect the nuances in approaches of the two services. See Table 10 for more detail on the background and approach of each service.

**Table 10:** Final recommendations for community palliative care, policy and practise

**1. Strengthening Early and Equitable Access to Palliative Care and End-of-Life** 1.1 Reduce professional gatekeeping by encouraging proactive identification and referral of patients who are approaching end-of-life and would benefit from holistic end of life care. 1.2 Ensure timely inclusion on the palliative care register, which should automatically trigger further service referrals to support patients and caregivers. 1.3 Encourage proactive identification and ensure access for non-cancer and underserved populations to palliative and end of life care services by improving integration of specialist palliative care, primary care, and community services.
**2. Enhancing Communication and Coordination Across Services** 2.1 Practitioners should develop competence in effective supportive communication to ensure shared understanding and high-quality care delivery. 2.2 Services should implement a locally agreed professional communication strategy to facilitate smooth referrals between providers and ensure patients and caregivers can navigate the system with ease. 2.3 A ‘named person’ or coordinator within services should oversee care provision and transitions, ensuring continuity of support.
**3. Expanding and Standardising Rapid Response Services (RRS)** 3.1 Ensure equitable access to RRS across all regions, reducing variability in provision (the “postcode lottery” of care). 3.2 Support 24/7 models of rapid response care where feasible, particularly recognising the vulnerability of patients and families at night. 3.3 Facilitate stronger integration between RRS, district nurses, general practitioners, and social care to ensure seamless transitions between services. 3.4 Where 24/7 care is not available, establish a dedicated phone line staffed by qualified clinicians to provide out-of-hours support, reducing inappropriate emergency service use.
**4. Supporting Informal Caregivers and Enhancing Bereavement Care** 4.1 Implement rapid assessment of informal caregivers’ needs, ensuring better access to resources and preparedness for end-of-life care at home. 4.2 Develop educational resources and training to support caregivers in managing the emotional and practical challenges of end-of-life care. 4.3 Ensure bereavement care is planned from the outset, with clear referral pathways to specialist support services.
**5. Improving Conversations around Death and Dying** 5.1 Promote public awareness campaigns to normalise conversations about end-of-life planning, reducing fear and stigma for all populations. 5.2 Improve routine data collection on patient journeys (e.g., out-of-hours contacts, emergency admissions, unanswered calls) to inform service improvements. 5.3 Increase awareness of palliative care services, particularly among those with non-malignant disease, by improving alignment between specialist palliative care and primary care services.
**6. Advancing Research and Service Evaluation** 6.1 Encourage an opt-out rather than opt-in approach to some palliative care research deemed to be lower risk, to enable larger-scale evaluations of service effectiveness. 6.2 Strengthen data sharing and service integration to build a more evidence-based, responsive palliative care system.

### PT2 service access – The importance of visibility and trust

Although feasibly patients can self-refer into the services, primarily, access is facilitated by referral from the district nurse (DN), who remains the first point of contact for service users. In addition, referral pathways can include GPs, palliative care teams and hospital discharge facilitators.

Continuity in care begins with timely access to services, yet many participants described gatekeeping by professionals as a barrier to RRS entry. The lack of early discussion about dying and reluctance to introduce palliative care services led to delayed transitions, reinforcing distress.

Rapid Staff Model B: *If you [discuss RRS] too early then I think you would probably worry the families a bit too much* (context)*, but then you don’t want it when they literally [are] in last days of life and they don’t know about this service* (resource) *and they get quite upset about that as well* (response), *not being told about this service that they could have used [it] maybes weeks before* (outcome). (RS9)

Gatekeeping from potential referral partners was reported as a barrier to access, particularly when referral pathways were unclear or when professional trust had not yet been established. In Model A, RRS staff reported their proximity improved service access, increasing visibility and trust.

Rapid Staff Model A: *the co-location of the nurses with the district nurses, particularly at the weekends* (context)*, is that real physical reminder of, […] if I'm stuck with anything, I can give them a call* (outcome)*. […] it's that visibility [that] is important for the access […]* (resource). (RS2)

In Model B, in contrast, the RRS had to undertake more active promotional work to achieve visibility.

Rapid Staff Model B: *I think the big challenge is people not knowing anything about us really* (context)*. […] GPs […] sometimes get us mixed up with Macmillan […]* (resource)*. And […] families do get a bit confused with the different services that are available* (response). (RS5)

Multi-disciplinary team (MDT) meetings were viewed as a vehicle to improve trust and awareness in both areas so improving opportunity for service access.

External HSCP Model A: *We do try and maintain the ability to […] retain continuity* (outcome) *as I say by the ways of having the meetings and having those good relationships with the district nurses and the palliative care* (resource)*.* (EX6)

Where the value of the RRS could be shared and trust built, opportunities to access the RRS could be improved.

External HSCP Model A: *the service has been really successful* (outcome) *[…] of all the calls, only 2% come from GPs. The rest have a 50/50 split from district nurses and patients and carers* (response)*, which I’m delighted about […] we didn’t design it for the GPs, we designed it for patients, carers, and actually our community staff* (resource)*. I think they realised what a good response and how helpful it is* (outcome). (EX3)

One caregiver explained how they accessed RRS through their GP, the GP recognising the requirement for specialist care. They also described how they would have experienced a difficult delay if they had relied solely on the DN’s.

Caregiver Model B: *the normal district nurses would have been weeks away, so the rapid response was initiated initially by the doctors* (context) *[…] The doctors realised my mother was coming up to 90 and it was the end-of-life treatment that you needed* (resource)*.* (FFC19)

Continuity provided for a sense of ‘being known’, which was important and highlighted as the crux of the palliative care model.

External HSCP Model A: *What's a positive outcome? […] We don't say […] did you have continuity?* (outcome) *We just talk about did you die at home? […] were your preferences met?* (outcome) *[…]. But I think that being known* (response)*, which is at the heart of palliative care* (resource). (EX6)

Caregivers in both models were reassured when they could rely on a service such as RRS to take the reins and provide continuity of care.

Caregiver Model A*: Just everything, she sorted* (resource). *[M]y husband want[ed] to stay at home* (context) *and [RRS nurse] said right we're putting the plan into action straight away* (response) *and she was on the end of a call* (outcome). (FFC14)

Once they had contact with the RRS, often patients and caregivers would want to maintain that relationship. One caregiver described the reassurance provided by continuity and accessibility.

Caregiver Model B: *there was that offer of we'll come back tonight* (resource) *[…] it was like […] somebody gave us a breath* (response) *because we were just working in the dark [before access] we didn't really know that that support [was available]* (context) *and it's a massive piece of support, and anybody who's never had to do that. I don't think they know […] [that Model B are] wonderful.* (outcome). (FFC6)

A short package of personal care is offered by the Model B service, providing further continuity. The value of this personal care service from Model B was addressed by a patient participant*.*

Patient Model B: *they gave me a good wash, and a spruce up, and did the bed for me,* (resource) *you know they were just brilliant, they more or less anticipate what you want* (resource) *[...] and it means a lot* (outcome) *when you don’t have to say to them, can you do this and can you do that, all the time* (response)*, nothing is any trouble to them* (outcome). (P1)

Similarly, a Model B staff participant described several differing service responses for differing requirements, all of which aim to reduce pain and unnecessary stress.

Rapid Staff Model B: *Some will say, “what do we do when it when [death] does happen?” We say, “don't worry about it. You don't phone 999 and you phone us (*resource) *[…] so they don't panic anymore* (response). *So, they know that they've got us from start to finish* (outcome). (RS7)

For caregivers, knowing who to contact and when could be confusing and it could lead to discontinuity or disconnection.

Caregiver Model B: *I didn’t fully understand people’s roles* (context) *[…] It was like, just somebody coming in. I didn’t fully understand it. And I didn’t understand the relationship between the district nurses and the [Model B] nurses* (response). *[…] I think a couple of times, I’ve gone through to the district nurses and then there had been no answer* (resource) *or I’d been in a bit of a panic, and I’d just rang directly to the [Model B] nurses* (response)*, […] Because that was the first number that was there* (resource). (FFC18)

Where continuity of care from a valued specialist can be accessed, relationships of trust and care could be developed.

External HSCP Model A: *I think trust is a is a big thing* (context) *and people are scared when it gets to out of hours and they will call on the person they trust* (response)*, or the service […] that they trust will respond* (response) *[…] you contact who you think you can get hold of […] regardless of the problem* (outcome). (EX5)

Several caregivers from Model B explained to us how the RRS improved the opportunity for dignity and perceived well-being of caregivers and the bereaved.

Caregiver Model B: *The smell wasn't so wonderful, and I don't know how to put this nicely, ‘cause it it's not nice is it death?* (context). *But it was nice to know* (response) *that when the undertakers came to take her, she was washed and clean* (resource) *[…] It’s massive that dignity and respect* (outcome). (FFC6)

Models A and B can be seen to be organised differently, in terms of an integrated person centred or adjunct relationship centred service (see below), and this leads to differences in modes of access and referral. Despite this, common factors could be seen across both models, with visibility and trust being important in both RRS for access, this is set out in PT2.1 and PT2.2 in Table 5, which are PTs that are generic across both models. Both services provide continuity of care which provided positive impacts through death and dying and into bereavement. How they do so, and the impact of the different service models on this, are identified in PT2.3 and PT2.4.

### PT3 timely response – reducing crisis and building confidence

Participants emphasised that a rapid and predictable response from professionals was essential to mitigate fear and uncertainty when caring for someone at the end of their life.

Rapid Staff Model B: *They've been discharged 11 o'clock at night, their loved one is near the end-of-life* (context), *and they are frightened, they’re kind of like a bit like a rabbit in the headlights* (response)*.* (RS6)

One RRS staff participant from Model B, described the value of attending homes ‘there and then’ where the household may be in crisis.

Rapid Staff Model B: *[W]here people haven't slept for hours [in] very stressful households* (context) *that they just want to hear that you'll help* (resource) *[…] I just say to them, yes, your district nurse is your key worker, […] but if you […] might not get a hold of the district nurse […] when the family needs help in palliative care, sort of crises, they do need help* (response), *and the last thing is you want somebody struggling for hours and hours on end* (outcome).* (RS8)*

One caregiver described the different means that they used to contact the RRS in a timely way and the value of that to them.

Caregiver Model B: *They just said, […] phone us night or day and […] we'll talk through what you need to do* (resource). *And then about four or five occasions, I would have phoned, and they would send somebody out who was qualified to give my mother some of the agitation drugs* (resource), *which was, […] absolutely great* (response).* [So, she could] settle down and [be] peaceful for the night* (outcome). (FFC19)

RRS staff were able to offer comfort to informal caregivers, providing reassurance and emotional de-escalation, while they undertook the difficult and extraordinary task of supporting death and dying at home.

Rapid Staff Model B: *Sometimes the families are frightened, even though they've been well prepared* (context). *[When] there's only hours left or could be anytime. Sometimes they still get that panic* (context). *[We] would give them support, we would say how normal it is for this to happen* (resource) *[...] We would never leave somebody, frightened and scared and panicked, because we are there for the family as well* (outcome). (RS6)

### PT4: 12 vs 24 h – the challenge of the night

Model A provides a specialist planned out-of-hours service 12 h a day 7 days a week (8am-8 pm) with cover from generalist services overnight (111- GP/DN) and Model B provides a specialist service 24 h a day 7 days a week.

Supporting death and dying at home can be challenging throughout the day and night, although night was a particularly vulnerable time.

External Staff Model A: ‘*It’s usually that people ring because they’re frightened* (context), *and they don’t understand what’s happening* (response). (EX1)

Rapid Staff Model B: *I think we expect a lot from families and carers* (context) *[…]. [P]eople are frightened by [death and dying]* (response) *and they're worried by it, and they don't know what's normal* (outcome). (RS6)

In Model A, where the 24/7 service was unavailable, a combination of daytime planning and general out of hours services were used.

Rapid Staff Model A: *As long as […] the more intense work on the day is done and they’re primed up that you know the documents are written up correctly, the drugs are there, the, you know the handovers are good, the communications good* (context) *there shouldn't be anything that is so desperate between 10:00 and 8:00 the next morning* (outcome). (RS2)

Yet, whilst the 12-h service was seen as perhaps practically and economically expedient, Model B staff had concerns about how much planning could happen.

*Rapid Staff Model B: in reality, does the pre-planning always take place like it should for the * [13]* hour service?* (context) *I don't know that it always does, you just need a bit of extra staff shortage* (resource), *[…] we are used all shifts. I think there's better communication with a 24-hour service* (resource). *There's more time for it* (context). *[…] I think it's reassuring to the patients to know that at any point of that 24 hour that they can ring if they need* (response). *And I think that massively cuts out their anxiety* (response). (RS8)

A staff member working alongside Model A, suggested that an out of hours specialist phoneline for caregivers to obtain overnight support would be valued and maintain continuity overnight.

External Staff Model A*: I do think that having someone on the end of the phone who is knowledgeable and […] I hesitate to say expert, because I don’t think this necessarily needs to be […] someone who is very specialised* (resource), *but I do think it needs to be someone who can provide support* (resource) *and reassurance* (response) *but also […] someone does need to be there if they need medication changes etc. in the middle of the night, because things […] happen in the middle of the night and we potentially can’t predict* (context). (EX4)

Overall, it was felt that some level of support was required through the night for service users to manage the challenge of the night. Ideally, this was a 24/7 service, although where this was not possible, collaborative working could cover some gaps. The final PTs for 12v24hr care, PT4.1 24 h Specialist Support and PT4.2 12 h Planned Specialist Support, can be found in Table 8.

### PT 5: beyond cancer care – addressing inequalities in access

The historical link between palliative care and cancer has meant that non-malignant conditions often faced delayed or inconsistent access to end of life care services such as RRS. Staff observed a persistent knowledge gap among referrers, particularly for conditions like heart failure, COPD, and dementia.

Rapid Staff Model A: *I think we've all had plenty of referrals where it's somebody who's been diagnosed with a cancer and they get referred to palliative care. And […], actually the cancer is the least of their problems, I'd have seen them for their heart failure two years ago* (context). (RS4)

One stakeholder from early information gathering stages described the “well-trodden pathway” of cancer diagnosis which can give patients, caregivers, and services more time to prepare for end-of-life:

Stakeholder Model B: *historically, palliative care and cancer are just interlinked as […] someone is going to die […] there is a big understanding, things are going to change, […] they need referrals and support, and it is a well-trodden pathway […] you are curative or palliative and that is made explicit to you*. *[…] Other diseases […] are palliative from diagnosis, but they don’t use that word* (context). (SH4)

The evolution of palliative and end of life care services, such as the RRS, can be problematic for timely transition for those with non-malignant conditions.

External Staff Model A: *When there’s a degree of uncertainty* (context), *it’s more difficult, consequently you need a bigger service* […] *being in the know and asking, and saying, “I want to be referred to palliative care” […] will only happen with certain groups of people* (resource). *Referral is where discrimination starts probably* (outcome). (EX4)

The importance of timely transitions was recognised by a caregiver from Model B, who shared the experience of rapid deterioration of her parent, which resulted in a referral. While they went on to describe receiving a high standard of care, that made a positive difference, she remained concerned about ‘right’ timings of referral into this care.

Caregiver Model B: *[brother] rang us, […] and he said, “me mam’s lost the use of her legs. She’s in bed and I’m going to ring the doctor”* (context) *and the doctor came and just said, “end of life care”* (resource). *[…] Now I look back and I actually think, maybe we should have tried to get my mam out of bed* (response). *[…] I feel as though sometimes she was a little bit condemned too early* (outcome). (FFC18)

The following caregiver from Model B had access to the service for their wife, who died with cancer, yet, in the past, when they had cared for their mother at the end-of-life with Alzheimer’s Disease, they had struggled to obtain any support.

Caregiver Model B: *I looked after my mam when she had Alzheimer’s for the last few years* (context). *And there wasn’t a fraction of the help that there is when somebody mentions cancer* (resource). *[…] There was nothing. I couldn’t get anything* (outcome). (FFC11)

Awareness and understanding of the services (and how to access them) were limited among prospective patients and caregivers, leading to uncertainty about available support and eligibility.

Caregiver Model B: *I always think [Model B] back to cancer* (context). *So, the thought of [Model B] doing what they did for us for end of life was unbelievable* (response). *[…] it wasn't cancer [that parent died from], so [we] try to share that message with everybody to say […] [Model B supports] more than cancer* (outcome). (FFC11)

The lack of recognition that non-malignant conditions do not preclude patients from end-of-life care, impacted continuity; not just in care delivery, but in who was even included in the system from the beginning. This led to the refined PT for this area, set out in Table 9.

### PT6: responsive skilled support – sustainable continuity

Continuity of care is often contingent upon the presence, capability, and support available to informal caregivers. Participants, both staff and caregivers, noted that without family or close friends providing informal support, it was significantly harder for patients to remain at home, regardless of service quality.

Rapid Staff Model A: *If you have a relative or a caregiver, you're more likely to stay at home for end-of-life care* (context). *The patients that I struggle to get to stay at home [are] those that don’t have anybody* (outcome). *[…] So, I could imagine you probably only really get those engaging with rapid response* (resource) *who have got somebody in the house to make those calls for them if they're deteriorating or unwell* (response). (RS11)

Informal caregivers often had to navigate practical and emotional challenges, with their capacity to provide care being shaped by multiple factors, including physical ability, personal resources, and relational dynamics.

Caregiver Model B: *My father was 91 and would have struggled to look after my mother in […] any physical way* (context). *[It] wasn’t an option [as] it was two males in the house* (resource)*. So, we couldn’t […] give me mum the care that she needed, privately* (response)*. […] I’m quite lucky because I’ve sort of semi-retired, so I took three months off to look after my parents* (resource)*. […] If I had not been able to do that, I don’t know where we would have been, to be honest with you* (response). (FFC19)

For some families, particularly those without an available caregiver, the feasibility of dying at home was limited.

External Staff Model A: *I don’t think you can die at home if you live by yourself* (context). *[…] Obviously people do, but, […] if it’s more complicated death […] we’re highly reliant on lay carers and family carers, and […] underestimate […] the need for them, and what [is] needed of them (resource), and what the result of that is [on] their bereavement* (outcome) *[…] positive or negative* (response)*.”* (EX4)

Additionally, informal caregivers often struggled with the transition from family member to full-time caregiver, sometimes finding the role emotionally and physically overwhelming.

Rapid Staff Model B: *All of a sudden, they turn in their roles into being nurses* (resource), *and that’s very difficult for them* (response)*.'Cause even though they love the person that they live with, it’s difficult for them to care for them* (resource) *when they feel out of their depth* (response)*.* (RS7)

Where informal caregivers were unable to meet the demands of home-based palliative care, the RRS played a vital role in bridging the gap by offering flexible, skilled, and timely support. Participants valued RRS interventions that provided both physical and emotional reassurance.

Rapid Staff Model B: *Patients say they want to be at home and they want to die at home* (context), *but I think that does come at a tremendous cost to families* (response) *[…] because […] even with lots of help going in, [with] carers four times a day* (resource)*, [...] [s]ome of the care agencies [...] [the patient has not] been washed* (outcome)*, [...] if the [care] staff are not experienced* (resource), *they're almost frightened to touch and to clean a dying person* (response). (RS8)

Where prescribing was available from the RRS staff, this provided a further layer of support, ensuring timely intervention and reducing unnecessary hospital admissions.

External HSCP Model A: *GPs are spread too thin* (context), *so […] you have to think about compensating for the fact that they won’t get through on 111, or if they do, there’ll be someone who doesn’t know them, or who is rushed off their feet* (resource)*, and whose knee-jerk reaction potentially is to admit someone to hospital* (response). *So consistently having someone who is Band 7 and can prescribe* (resource) *[as] who cannot prescribe is not rushing round trying to get a GP to prescribe something* (outcome) *is a sensible way forward.* (EX4)

Ensuring relational continuity between caregivers and RRS staff mitigated fears of ‘not doing it right’ and built trust that care would be appropriately managed.

Stakeholder Model B: *Sometimes carers will be reluctant to phone as they worry, we will come in and say [we] have to take [the] patient into hospice as they aren't coping* (response)*. So, [we need to] have the conversation earlier* (context), *dealing with the emotions of dying, and then on top of that the worry about doing it well enough, and the patient worrying about being a burden* (resource). (SH2)

Ultimately, flexible, responsive, and skilled support facilitated holistic and person-centred care, ensuring caregivers felt empowered rather than overwhelmed. This is outlined in the refined PT for this area in Table 9.

Across all programme theories, continuity of care emerged as the defining factor in positive end-of-life experiences at home. Whether through early and reliable service access, speed of response, 24-h availability, inclusive care, and flexible caregiver support, participants consistently suggested that ‘being known’ reduced fear, improved decision-making, and enhanced emotional well-being. Conversely, discontinuity whether through gatekeeping, professional distrust, service fragmentation, or caregiver strain, led to unnecessary distress, delayed care, and, in some cases, avoidable hospital admissions.

To support translation of these findings into policy and practice, we developed a set of actionable, theory-informed recommendations drawn directly from the refined programme theories; these are presented in Table 10.

## Discussion

This study highlights the crucial role of continuity of care in facilitating positive end-of-life experiences at home. Across all refined programme theories, participants consistently emphasised that ‘being known’ by RRS staff fostered trust, reduced distress, and improved decision-making. The findings demonstrate that timely and responsive care significantly enhances both symptom and emotional management, providing person-centred care and ensuring that caregivers feel supported and confident in providing end-of-life care. This aligns with existing research highlighting the crisis points in outpatient palliative care, where caregivers often feel overwhelmed without timely professional support [34].

As our previous paper [33] highlights, the potential reticence of informal caregivers or patients to discuss or recognise end-of-life was an early access barrier. This reticence was sometimes alluded to in participants’ accounts and is supported by prior research on the delayed acceptance of end-of-life among patients and caregivers [35]. Similar dynamics have been identified in qualitative research exploring how uncertainty about what to expect in the final days, shaped by unspoken norms and limited preparation, can inhibit engagement and emotional readiness at the end of life [36]. Previous research [2, 37] emphasises that increasing public awareness and professional confidence in discussing death improves service engagement and quality of life at end-of-life. Late transitions into palliative care, often due to gatekeeping and reluctance to discuss death, can limit opportunities for early intervention and planning. Furthermore, caregivers who experience distress and regret in the lead-up to a relative’s death may struggle to fully engage with available support services [38]. Addressing these barriers through proactive engagement and open communication is essential to improving palliative care accessibility.

The provision of a timely response to crises and calls for help, particularly during the night when patients and caregivers felt most vulnerable, mitigated fear and uncertainty. Variability in service models impacts the degree of continuity and holistic support, with 24/7 RRS models (Model B) providing more sustained relationships than limited-hour services (Model A). Further, non-cancer patients experienced more difficulty accessing palliative services, reinforcing existing health inequalities. Research highlights how interdisciplinary approaches, particularly those that focus on establishing relational trust, improve the effectiveness of palliative interventions [39]. Regardless of these accessibility barriers, informal caregivers bear significant burdens while supporting end-of-life at home; they require skilled support to navigate their role transitions and sustain changing and challenging responsibilities. When they were able to access continuity of care from the RRS, this provided a practical and emotional boost, offering stability in an otherwise unpredictable and worrisome time, helping to smooth transitions.

### The importance of transitions through care

To better understand these findings, we embedded transition theoretical thinking across the study. To better understand these findings, we embedded transition theoretical thinking across the study. Meleis'Transitions Theory [27] helped us conceptualise how patients and families navigate multiple transitions at the end-of-life, these include, health status changes, shifting familial roles, and movement through services [29]. A qualitative meta-synthesis of transitions in palliative care highlights that these role shifts are not only medical but deeply personal, emotional, and relational, requiring structured support to avoid distress and fragmentation [40]. The transition into palliative care is often delayed due to uncertainty about prognosis, professional reluctance to discuss dying, and public fears about what palliative care might entail [41] [42]. Following this, caregivers face the transition from family member to caregiver, which can be emotionally overwhelming, particularly without structured support [14]. Finally, families navigate the transition through active dying and into bereavement. Research shows that when caregivers feel unprepared for these transitions, they are more likely to experience distress and unresolved grief [43]. In contrast, the presence of trusted and familiar RRS staff during these transitions can positively shape how families experience loss, grief, and ‘closure’ [44].

By embedding relational continuity, RRS staff not only provide symptom management but also help patients and families navigate these role changes, emotional shifts, and existential concerns. In contrast, fragmented care leads to disrupted and destabilised transitions, distress, and potential hospital admissions [42]. Beyond practical support, continuity of care plays a vital role in structuring the dying process; familiarity and recognition from caregivers create a sense of stability, dignity, and possible meaning at the end of life [44].

In this study, participants described how RRS staff functioned as guides during this uncertain and often fearful time, providing reassurance during crises. Knowing who to call, particularly in Model B, where they could access support 24/7, reduced fear and uncertainty. A sense of being known, where RRS staff remembered patient and family histories, preferences, and fears, created a relationship of trust. In contrast, where services were inconsistent, participants described feelings of abandonment, confusion, and distress, which aligns with wider literature on the psychological impact of fragmented care in life-limiting illness [45].

### The future of community palliative care

This study contributes to discussions about health inequalities in palliative care. Non-cancer patients experience barriers to service access, often due to uncertainty around prognosis and healthcare professionals'reluctance to introduce palliative care early [46]. Furthermore, limited 12-h services disproportionately impact those with fewer informal care resources; night-time is known to be a particularly distressing and vulnerable period [47]. Recent research underscores the importance of skilled communication in end-of-life care, emphasising that professional guidance can help caregivers manage the psychological and existential aspects of providing care [48]. As other recent research has found [49, 50], caregivers experience significant anxiety and strain when caring for someone at the end-of-life, and support services are therefore highly valued. Where RRS is available and accessible, they can contribute to compassionate communities and public health approaches that bridge gaps in care [51].

Home death is not just about preference; it requires an ecosystem of care, including specialist services, trained caregivers, and broader social support [51, 52]. Improving death literacy, strengthening community support networks, and embedding relational care models into existing services are essential steps towards equitable and effective end-of-life care [1]. Our study highlights the urgent need for policies and service models that ensure early, proactive engagement with palliative and end-of-life care within a broader social support system. This requires reducing gatekeeping barriers and increasing professional confidence in initiating conversations about death [41].

The RRS provides an invaluable safety net, but their effectiveness is hampered by inconsistent availability, underfunding, and professional distrust. Supporting death and dying at home requires more than the specialist skills of the RRS staff; it requires relational continuity, social trust, and holistic support. When implemented effectively, RRS can bridge the gaps between generalist and specialist care, transforming death at home from a fearful and isolating experience into one that is supported, traversable, and dignified.

These findings informed a set of evidence-based recommendations for the design and delivery of rapid response services, particularly in relation to referral pathways, continuity, and out-of-hours support. These are summarised in Table 6 and reflect the refined programme theories developed in this study.

### Limitations and future research

Conducting research on end-of-life care presents inherent challenges, particularly in recruitment. Despite efforts to engage district nurses, nursing home staff and ambulance services, participation was limited. Similarly, while our gatekeepers facilitated caregiver recruitment, some found participation emotionally overwhelming or burdensome, highlighting practical and ethical constraints in end-of-life research [53, 54].

The study sample predominantly reflects white British, middle-class participants, aligning with existing research that suggests inequities in access to palliative care services [46]. Future research should actively engage underrepresented communities through peer research models and community outreach [55, 56].

Despite these limitations, the realist evaluation approach provided depth of insight into the generative mechanisms and key contexts underpinning community end-of-life care. In line with realist evaluation methodology, we aimed to produce theoretically generalisable findings through mechanism-based middle-range theorising, which supports both explanatory power and contextual sensitivity [57].

The contrasting programme theories observed offer a valuable basis for future inquiry. Building on our realist findings, a next step could involve working with service users and professionals to co-design an adaptive model that draws on the complementary strengths of each approach. Such work could retain a realist lens, exploring how and for whom key elements work, while also engaging with further questions of feasibility and scalability relevant to implementation in real-world contexts.

## Conclusions

This study used a realist approach, supported by transitions theory [27], to demonstrate that continuity of care is a defining factor in enabling positive end-of-life experiences at home. Rapid response services (RRS) play a crucial role in maintaining stability, trust, and timely intervention, providing essential reassurance to both patients and caregivers. Across all programme theories, relational continuity emerged as a key enabler, reducing fear and emotional distress while fostering trust in professional care. The 24/7 availability and broad scope of care offered in Model B led to greater confidence and reduced uncertainty, while Model A, relying more on pre-planning and professional coordination, still demonstrated benefits but with limitations in overnight support.

Despite growing recognition of home death as a preferred option, systemic barriers such as late service transitions, professional gatekeeping, and inequitable access to RRS continue to create disparities in end-of-life care. The findings emphasise the importance of timely, proactive engagement with palliative care and the need for integrated service models that support both patients and caregivers through the complexities of home-based dying. Future policy and practice must prioritise relational continuity, reduce access barriers, and ensure that RRS are embedded within a broader compassionate community framework, enabling dignified, well-supported deaths at home.

## Supplementary Information

Supplementary Material 1

Supplementary Material 2

Supplementary Material 3

Supplementary Material 4

Supplementary Material 5

## Data Availability

Data are held securely by the research team and may be available upon reasonable request and with relevant approvals in place.
