# Supplementary material for: ‘People ring because they’re frightened’: findings from a realist evaluation on the impact of timely responsive care at home at the end of life
Source: BMC Palliat Care. 2025 Jul 14;24:199. doi: 10.1186/s12904-025-01826-y (PMC12261533; doi:10.1186/s12904-025-01826-y)
Supplement: Supplementary file 2 — Supplementary Material 2 [file 12904_2025_1826_MOESM2_ESM.docx]

**RRS - IPTs and CMOs**

This document lists the 8 different IPTs, their initial key points we generated for clarity for group discussion, followed by different CMOs which have been developed through early analysis and revision of theories. The final agreed refined PTs are outlined at the end of each sub-section.

**IPT-PT Overview**

| **IPT Title** | **PT Title** |
| --- | --- |
| **IPT1** Communications | **PT1** Skilled Communication |
| **IPT2** Values | Ended – data contributed towards refinement of PT6 Responsive Skilled Support and PT5 Beyond Cancer Care |
| **IPT3** Access | **PT2** Service Access |
| **IPT4** Diverse Needs | **PT5** Beyond Cancer Care |
| **IPT5** Geography and Community | Ended – insufficient data to test the theory |
| **IPT6** 12v24hr | **PT4** 12v24hr |
| **IPT7** Timeliness | **PT3** Timely Response |
| **IPT8** Who | **PT6** Responsive Skilled Support |

# **IPT1 Communications**

If patients and their families have the opportunity (context) to receive open and timely communication about the RRS at the end of life (what it is, what it offers, when and why) (resource), they can make an informed decision about death and dying at home (reasoning) and so will self-refer to the RRS (outcome-1), and/or post-referral (self or DN/GP) use the RRS, as opposed to another emergency service, in times of need (outcome-2), and admissions to hospital at end of life will be reduced (outcome-3).

**Key point:**

Open and timely communications, that are clear and appropriate, support people to engage with the RRS and provide information as service users move through transition(s) into end-of-life care and through end-of-life care period into death/post-death period.

**CMO COMMUNICATION: HONESTY**

**CONTEXT/S:** service users are **unaware** of gravity or reality of the situation- i.e., that the patient is at end of life, AND/OR service users are **unprepared** for awareness of the gravity or reality of the situation- i.e., that the patient is at end of life

**MECHANISM/S:** the staff use their time/flexible verbal/non-verbal communication skills to provide knowledge and psycho-social support that is appropriate to that service user (i.e., may or may not use 'death'- and cannot give an 'expiration date' as RS4 explained) (costs: expert skills/knowledge/training)

**OUTCOME/S:** ***positive***- service users better understand the situation and can prepare and adapt, their expectations are better managed, and consequently they are reassured and better 'prepared' to care (benefits: patients feel reassured/empowered, positive impact on well-being). ***negative***- this cannot always be managed (as RS5 syringe driver) although will try best to manage.

**CMO COMMUNICATIONS: CHAOTIC AND TURBULENT COMMUNICATIONS**

**CONTEXT/S:** managing expectations and/or understanding different needs and concerns of a full household is difficult at the end of life - particularly where situations, and so 'preparedness', may change over time (costs: staff having time prior to visits e.g., read information/preparation, multiple visits/contact)

**MECHANISM/S:** staff use their time/flexible verbal/non-verbal communication skills to support difficult household communications, managing tension and competing needs and desires, supporting all to be heard and understood (costs: staff training/time; benefit: families being heard/part of decision-making process)

**OUTCOME/S:** service users better understand the situation and can prepare and adapt, their expectations are better managed, and consequently they are reassured and better 'prepared' for whatever comes next - be that end of life at home or in hospice (costs: reduction in unplanned care e.g., emergency admissions, ooh GPs etc; benefits: increased reassurance/interactions/experience/empowerment)

**CMO COMMUNICATIONS ‘EXPERT COMMUNICATIONS’**

**CONTEXT:** Because of low death literacy and the intensity of the emotional and physical experience of supporting someone to die at home … (costs: staff having time prior to visits e.g., read information/preparation, multiple visits/contact, increased costs for more qualified ‘expert’ staff (i.e., higher salary/training costs for grade 7) and/or additional HCA staff)

**MECHANISM/S:** repeated expert communications are required on an 'as and when required' basis, considering the 'triad' of experts involved which leads to appropriate and timely sharing of information that helps manage expectations (costs: training, multiple visits/travel and multiple staff)

**OUTCOME/S:** this leads to positive outcomes for service users/service (costs: reduction in unplanned care e.g., emergency admissions, ooh GPs etc; benefits: increased reassurance/interactions/experience/empowerment)

**CMO COMMUNICATIONS ‘STAFF AVAILABILITY/MODELS/TIME’**

**CONTEXT/s:** complex (chaotic, concerned, distressed households).

**MECHANISM/s:** two members of staff in DRRS- can manage multiple aspects of care and multiple household members at once - so can provide pain relief + communicate for reassurance to FFC simultaneously (costs: additional cost of two staff members rather than 1 e.g., NRRS)

**OUTCOME/s:** managing multiple aspects of care with positive outcomes for patient and FFC (Costs: for NRRS – cost of providing multiple aspects of care and outside 8-7pm, what cost and to whom? Benefit: less calls to/input from GP/DN; Benefits: more streamlined/specialist care, reduction in the use of emergency/ooh services and hospital admissions)

**CONTEXT/S:** where there is time available for the RRS staff to ascertain need and appropriateness (costs: staff having time prior to visits e.g., read information/preparation, multiple visits/contact)

**MECHANISM/S:** staff can 'teach' or provide knowledge and care activity to FFC at home (benefits: increased reassurance/empowerment)

**OUTCOME/S:** positive outcomes for patient (e.g. comfort from mouthcare) and for FFC (e.g. inclusion and understanding leading to improved well-being in short and longer term) (costs: increased time spent with patients/carers; benefit: families being reassured and confident to provide care/positive interactions with RRS)

**CONTEXT/S:** where there is time available for the RRS staff to ascertain need and appropriateness (costs: staff having time prior to visits e.g., read information/preparation, multiple visits/contact; staff training/awareness)

**MECHANISM/S:** staff can build a relationship with patient and FFC to understand differing needs and so provide a culturally sensitive package of care (benefits: increased reassurance/empowerment)

**OUTCOME/S:** respect, engagement, positive outcomes from completing appropriate rituals (costs: increased time spent with patients/carers, training/awareness; benefit: families being heard/reassured/understood and positive interactions with RRS)

**Refined Programme Theory:**

**PT1 Skilled Communication**

Dying at home can be a difficult and challenging time for patients and informal caregivers, who may have had no or limited exposure to death and are fearful of it **(context).** When experienced nurses with compassionate communication skills can be flexible with time spent with families and caregivers **(resource)** this increases understanding and provides comfort and reassurance to patients and caregivers **(response),** who then feel a sense of preparedness and empowerment to manage dying at home **(outcome)**, which can improve the ability to appreciate this important and valuable final time together **(outcome)**.

# IPT2 Values

Rapid Response Services display implicit and explicit values and expectations to potential service users (context-resources), where there is some misalignment of values and expectations for patients/carers, they feel fear/dislike/distrust and/or that they will be poorly judged by the service (mechanism-reasoning), and this leads them to choose not to use the service and/or seek assistance elsewhere, which could result in more hospital admissions (outcome)

**Key point:**

Expectations of service and service users may be misaligned (fear of judgement/lack of fit/rejection). This is about cultural awareness and appropriateness of RRS staff and whether there is something in the way the service is set up, promoted, or delivered that works to (unintentionally) exclude.

**CMO VALUES ‘EXCLUSIVE EXPECTATIONS FOR BEHAVIOUR’**

**CONTEXT:** The RRS has implicit expectations of ‘appropriate’ behaviour/s around death and dying

**MECHANISM/S:** When these are not met or are deviated from there is a tension between the service and the service user which is unintentionally excluding and so service users feel the service is not for them (or will be afraid of the service) (costs: reduction in service use/negative interactions/experiences)

**OUTCOME/S:** The RRS is not used (as often/frequently) and/or when it is used the service user experience is impacted (costs: reduction in service use/negative interactions/experiences, increase in use of other/emergency services e.g. 999/ooh GP etc and hospital admissions)

**CMO VALUES ‘LOSING CONTROL’**

**CONTEXT:** patients and their supporters are afraid of losing control, losing privacy, and pain (costs: staff training/expertise, time spent understanding/responding to concerns)

**MECHANISM/S:** RRS staff provide positive communication and inclusive conversations, over multiple interactions, and are adaptive in the service they offer, these reassure service users and maintain awareness and acceptance (costs: increased time/multiple visits/travel; training; benefits: patients/carers feel reassured/experiences valued)

**OUTCOME/S:** patients’ pain is managed and supporters understand what is happening and what may come next and feel in control of situation (costs: reduction in use of other/emergency services & hospital admissions; benefits: increased interactions/experiences with RRS, reassurance and confidence)

**CMO VALUES ‘NOT LIKE ME’**

**CONTEXT:** The RRS staff and service model do not look/sound/apply to me

**MECHANISM/S:** I will not be understood, I will be judged, RRS does not apply or suit our family and our needs (costs: potential increase in use of other/emergency services, hospital admissions)

**OUTCOME/S:** service will be avoided, rejected, or service users may have a poor experience (costs: potential for greater use of other less specialised and/or emergency services (e.g., 999/hospital admissions) and less positive experiences of RRS/feeling unsupported and increased patient/carer burden/distress)

**CMO VALUES ‘INCLUSIVE ACTION’**

**CONTEXT:** DRRS undertake inclusive promotion activities through wide distribution of leaflets (costs: staff training/awareness/communication; promotional materials produced in different languages)

**MECHANISM/S:** this allows for those who may be excluded from other services and so unable to access through referral to access (costs: potential increased use of other/emergency services)

**OUTCOME/S:** potential engagement through the service for those who may be excluded from other mainstream services (benefit: proportion of people from different backgrounds/circumstances accessing intervention; positive experiences of RRS including feeling valued and respected)

**CONTEXT:** The RRS undertake training and regular informal team discussions to maintain cultural awareness, reducing potential conflict and unintentional judgement, recognising diverse needs of their wider community (costs: staff training/awareness, range of leaflets, time spent)

**MECHANISM/S:** So, when they attend service users homes they are flexible to differing requirements and needs and promote inclusion (benefits: positive experiences of RRS, feeling valued and respected)

**OUTCOME/S:** service engagement is encouraged (costs: staff training/awareness, time spent; benefits: proportion of people from different backgrounds/circumstances accessing intervention; positive experiences of RRS including feeling valued and respected)

**CONTEXT:** The RRS has multiple opportunities to meet with service users in the end of life period (costs: time spent/multiple visits and travel; training/awareness)

**MECHANISM/S:** RRS staff and service users can have multiple conversations about providing an appropriate service that meets different cultural expectations and needs and avoids assumptions, which leads service users to trust in the service (costs: time spent/multiple visits; staff training/awareness; benefits: increased positive experiences, feeling valued and respected/ increased trust)

**OUTCOME/S:** service meets user cultural needs and so positive outcomes for service users (benefits: proportion of people from different backgrounds/circumstances accessing intervention; positive experiences of RRS including feeling valued and respected)

**CMO VALUES ‘EXCLUDING REQUIREMENTS’**

**CONTEXT:** The RRS attends to service users at end of life in the community with symptom management and other aspects of care (costs: multiple visits / attending to additional care needs)

**MECHANISM/S:** yet to sustain/manage at home the service user also needs at-home resources heat/power/appropriate bed/carer/home which all require a certain level of economic/financial security where these are not available the ability to be at/manage at home may be reduced (costs: increased ‘burden’/use of personal resources)

**OUTCOME/S:** the service cannot be accessed or engaged with (costs: reduction in patients/carer access to services, increased burden on personal resources; potential increase in use of other/emergency services and hospital admissions)

**Refined Programme Theory:**

**Ended** – data contributed towards refinement of PT6 Responsive Skilled Support and PT5 Beyond Cancer Care

# **IPT3 Access**

When pathways into the Rapid Response Service (RRS) are clear, transparent, and unconstrained to all that could benefit from the service (context), patients are able to access the immediate care that RRS offers if necessary (resource) and therefore feel confident to provide care at home (reasoning) which leads to less emergency calls (outcome 1) and more home deaths (outcome 2).

**Key point:**

Although can feasibly self-refer, primarily access into the RRS is by referral by DN (most likely) or GP, palliative teams, discharge- this means those referral pathways need to be appropriate and unconstrained.

**CMO ACCESS ‘ON THE BOOKS’:**

**CONTEXT:** a patient is required to be ‘on the books’ of another service to effectively access this service, e.g. the planned palliative service **(+/ they need to be identified as at end of life** and other services have accept/agreed with this diagnosis/status) (costs: increased time/pressures to engage/enrol patients into the services & communicating with other services, potentially much later than ‘needed’)

**MECHANISM/S:** referral pathway into the RRS from the other service/s are then clear/constrained (costs: time spent trying to access/communicate with (multiple) services)

**OUTCOME:** access is hindered/possible and service is un/utilised (costs: increased use of other/emergency services and potential increase in hospital admissions; patients/carers feeling unsupported/unheard)

**CMO ACCESS ‘SELF REFERRAL’**

**CONTEXT:** both services *feasibly* accept self-referral (costs: potential increase in self-referrals and how to prioritise; benefits: patients feel reassured there is a service that they can self-access)

**MECHANISM/S:** services can, and sometimes are, promoted in a manner that allows/encourages direct access to the rapid service by service user without use of another service as a referral partner (yet this appears unwanted/undesirable in some transcripts) (costs: increased self-referrals & need to prioritise/manage caseloads & signpost to other services) 
**OUTCOME:** ‘appropriate’ referrals are reduced and boundaries between services are transgressed with complications for service/service user and ***trust between services is maintained*** (costs: increased use of other/emergency services and/or hospital admissions, patients/carers feeling discouraged/frustrated; carer/patient time spent trying to access/communicate with services (e.g., phone calls) and less positive experiences of RRS)

**CMO ACCESS ‘WHOSE PATIENT?’**

**CONTEXT:** the RRS offers ***recognised*** ***specialist*** palliative care in the community at end of life, which other services are aware of, that can be accessed by referral from other services (costs: numbers referred/resources to provide services for all, ‘inappropriate’ referrals; benefits: appropriate specialist community care for patients/carers needs)

**MECHANISM/S:** the speciality/expertise/addition of the palliative care nurses/HCAs is trusted by other services so they are willing to refer as they remain the ‘key worker’ or keep ‘their patient’; ***co-location of services/staff can work as a positive mechanism to encourage knowledge, trust, and information sharing*** (costs: time/resources (& personal) spent maintaining positive relationships/collaborations; benefits: ‘clear’ referral pathways/communications maintained; ‘streamlined services’ can reduce stress/burden on patients/carers e.g., better communication/flow/less calls and/or time spent communicating with ‘different but linked’ services I.e., planned and unplanned care)

**OUTCOME:** access into the service is not blocked by a gatekeeper concerned their will lose their patient/patch/authority (benefit: increased patients/carers being able to access the intervention)

**CMO ACCESS ‘STICKING PLASTER’**

**CONTEXT:** when the boundaries of the service are in some way unclear AND/OR the boundaries and availability of other services are unclear (costs: potential for ‘inappropriate’ and/or increased referrals and the need to prioritise care and/or signpost; patient/carer frustration and feeling unsupported/confused)

**MECHANISM/S:** potential service users will mis/use the service and place inappropriate calls mis/directing staff time AND/OR not contact the service when they need to and should do as they fear they are a burden or are not sure that they can (costs: increased pressure on services to respond and/or signpost elsewhere)

**OUTCOME:** reduction in appropriate service capacity AND/OR poor outcomes for potential service users as they elect not to contact when require support (costs: increased pressure on services; patients/carers feeling unsupported/frustrated and potential increase in other/emergency services and/or hospital admissions)

**CMO ACCESS ‘THERE AND THEN’**

**CONTEXT:** Rapid team/service has clear relationships and boundaries with other services and this is communicated to all parties including service users: RRS is provision of ***end of life care at home*** (costs: relationships/maintaining communication requires time)

**MECHANISM:** Rapid respond to crisis situations, at home, not resolvable elsewhere, in a rapid manner ***with appropriate shared information*** (benefits: carers/patients receive timely/appropriate care with less time resources e.g., making calls)

**OUTCOME:** rapid respond to appropriate crisis calls with full information sharing and support from other services – ultimate positive outcome for services/service users (costs: maintaining relationships/communication requires time; benefits: patients/carers receive timely/appropriate care with reduced time spent communicating with different services)

**Refined Programme Theory:**

**PT2 Service Access**

Potential services users require straightforward and clear access to specialist palliative care at the end of life in the community, this could be through **integrated** or **adjunct** services **(context)**, by providing proactive information sharing within and between acute and primary care (e.g. shared information systems or proactive action by staff to share information) **(resource)** rapid response services can overcome gatekeeping barriers **(response)** and improve potential to service access **(outcome).**

**PT2.1 Gatekeeping and Professional Trust – Model A**

Model A offers an **integrated** recognised specialist palliative care service in the community at end of life (e.g., integration with the acute sector and primary care, including the DN service who are the first port of call) (**context**) which allows for formal and informal information sharing and working practices (e.g., co-location with acute sector and DN’s, shared SystmOne, and attendance at MDT where necessary) (**resource**). Shared information systems support recognition of the boundaries, roles, and speciality of the service increasing partners awareness, confidence, and professional trust **(response)** who, reassured, will be more likely to refer increasing opportunity to access the service **(outcome).**

**PT2.2 Gatekeeping and Professional Trust – Model B**

Model B offers an **adjunct** recognised specialist palliative care service in the community at end of life (e.g., offered alongside the DN service, who are the first port of call) (**context**) which allows for formal but restricted information sharing (e.g., facilitated through shared SystmOne access, outreach activity to engage with primary care, and attendance at MDT where invited) (**resource**). Proactive action by staff supports recognition of the boundaries, roles, and speciality of the service increasing partners awareness, confidence, and professional trust **(response)** and so, reassured, will be more likely to refer increasing opportunity to access the service **(outcome).**

**PT2.3 Continuity - Model A**

Model A provides a **person-centred approach** (e.g., focus is on the patient’s needs and wellbeing and interactions with other services but not as focused on caregivers) **(context)**. Where services are aware that patients have significant palliative care needs, they have access to a planned palliative care service and an adjunct rapid response service that can respond to the urgent and out of hours needs of the patient **(context)**, This service provides valued specialist care and works to maintain relationships between different teams and organisations to provide continuity of care **(resource)** until the patient dies. This develops relationships and supports caregivers to provide home care **(reasoning)**, with positive impacts on patient and caregiver well-being **(outcome)** but doesn’t provide support for death and beyond for the carer **(outcome)**.

**PT2.4 Continuity – Model B**

Model B provides a **relationship centred care approach** (e.g., incorporating the needs and well-being of not only the patient but also of their significant others, and prioritising relationships between professional staff) **(context)**. It provides specialist and holistic care underpinned by a palliative ethos, which leads to a robust continuity of care to the patient and caregiver, throughout the patient and carer journey (including at death, post-death, and bereavement) **(resource).** This will lead to relationships of trust and care **(reasoning)** with positive impacts on patient and caregiver well-being and patient dignity **(outcome)**.

# **IPT4 Diverse Needs**

Historically, community palliative care services were developed to support patients with cancer to die at home (context), now guided by the GSF and ACP (resource), professionals can demonstrate understanding of, and sensitivity to, the diverse needs of individuals (reasoning), and so appropriate referrals are made for those approaching end of life, regardless of diagnosis (outcome), but societal norms can stop people from engaging (context)

**Key point:**

How/does pain or multi-morbidities or frailty or dementia impact on service use?

**CMO DIVERSE NEEDS ‘SERVICE USER ACCEPTANCE’**

**CONTEXT**: Different disease trajectories can make prognosis and end of life difficult to predict and historically palliative/eolc has been more associated with cancer care (costs: increased care for ‘other conditions’ and/or multiple time periods depending on prognosis/need)

**MECHANISM/S:** patients may rally, or have been at ‘end of life’ previously yet appeared to have recovered, this leads to patients/supporters being unprepared to accept end of life care and reject the service/have problematic relationship with the service/attempt to use another service such as 999/111

**OUTCOME/S:** this can cause discomfort/distress for patient/supporters and/or increase likelihood of unnecessary hospital admission (costs: potential increase in utilising other/emergency services and/or more hospital admissions; negative experiences of RRS/less likely to access again, increased patient/carer distress, increased ‘burden’ on family)

**CMO DIVERSE NEEDS ‘DIFFICULT PROGNOSIS’**

**CONTEXT:** Different disease trajectories can make prognosis and end of life difficult to predict and historically palliative/eolc has been more associated with cancer care (costs: patients/carers may not access the intervention and feel unsupported, additional ‘costs’ on carers/families to provide own care)

**MECHANISM/S:** better use of GSF/ACP and interaction/knowledge of the RRS means GP/DN will refer (earlier) to RRS leading to access of service by users in good time

**OUTCOME:** this will improve experience for staff/service user (benefits: increased patient/carer access to the service, feeling supported; potential decrease in utilising other/emergency services in times of need and family/support networks)

**CMO DIVERSE NEEDS ‘BEYOND CANCER CARE’**

**CONTEXT:** As end of life care in the community was previously closely associated with cancer care there are still remnants of this concept/understanding in the population (costs: patients/carers not accessing the intervention)

**MECHANISM:** without active service promotion there are lower levels of awareness and subsequent use of RRS by those who have non-cancer diagnosis (costs: promoting the services, dealing with potential increase in demand/resources)

**OUTCOME:** service goes un/accessed (costs: potential increase in use of other/emergency services, patients/carers left unsupported)

**Refined Programme Theory:**

**PT5 Beyond Cancer Care**

Uncertainty around prognosis for non-malignant disease, as well as general fear and misunderstanding around palliation and end of life care (and their differences) in the wider population, can lead to a lack of awareness of, and timely transition into, palliative and end of life care **(context).** Well-funded, accessible services, rooted in primary care rather than aligned to the acute sector **(resource),** increase awareness, and improve access **(response),** leading to improved care for all patients and caregivers **(outcome)** and reduced hospital admissions **(outcome)**.

# **IPT5 Geography + Community**

Typically, older population profiles mean that living and dying in rural areas is by no means a homogenous experience (Hospice UK, 2021). The geographical spread of patients in rural areas is vast, meaning that a one-hour response is not always feasible (context), therefore when there is no timely response from RRS (within one hour) (resource), and family carers feel unsupported and unconfident to provide end of life care (EoLC) to their family member (reasoning), this leads to a higher likelihood of emergency phone calls and admissions (outcome).

**Key point:**

Could those in more rural areas be excluded from the service by their geographical position altering service/service response times?

Is collaboration (community) particularly important here?

**CMO GEOGRAPHY RURAL EXCLUSION**

**CONTEXT:** Potential RRS users in rural locations face multiple potential inequalities (costs: increased time/travel for services, prioritising patients, patients/carers feeling unsupported & increase frustration/ distress/burden)

**MECHANISM/S:** exclusionary factors include age, travelling difficulties, and a lower number of (specialist) services available

**OUTCOME/S:** services go un/accessed (costs: potential increase in use of other/emergency services and/or hospital admissions, patients/carers feeling unsupported; potential increase in deterioration before service is accessed, patient/carer time/travel costs to access services, potential costs to ‘other’ patients/carers (e.g., time/resources re-directed))

**CMO GEOGRAPHY RURAL COMMUNITY**

**CONTEXT:** Potential RRS users in rural communities have close community links with their local services (benefits: patients/carers feel supported and have access to appropriate/known care)

**MECHANISM/S:** established relationships with local GPs/DNs who are well known to service users mean service users will elect to use these services before RRS (benefits: reduction in staff time/travel; potential to re re-directed to other/more patients)

**OUTCOME/S:** services go un/accessed (costs: potential reduction in access to more ‘specialist’ eolc, potential increase in accessing ooh/emergency services and/or hospital admissions, patients/carers feeling unsupported)

**Refined Programme Theory:**

**PT Ended** – insufficient data to test the theory

# IPT6 24v10hr

Previous research (Dalkin et al, 2018) indicates that family members feel less supported and more vulnerable at night (context), where the RRS provides 24 hours care (as opposed to 10 hour care model) (resource), family carers feel confident to provide end of life care (EoLC) to their family member at home in the knowledge that they can access care overnight (reasoning), this leads to a reduced likelihood of emergency phone calls and admissions from a 24 hour service, as opposed to a 10 hour service, and so the patient preference for a home death is met (outcome).

Rival: 10hr services are aware of the limitations of their provision (context) and therefore work proactively to ensure planned care that equips carers with strategies (resources) to make sure they can cope overnight (reasoning) resulting in less emergency admissions and more home deaths (outcome).

**Key point:**

Does the ability to access a service over a 24hr period reassure patient/caregiver during the day and night as they know they have access during “the ordeal of the night” or can planned day services overcome this?

**CMO 24v10hr ‘THE “ORDEAL” OF THE NIGHT - 24hr CARE**

**CONTEXT:** As Dalkin (2018) describes, there is a vulnerability to the night, described as an ‘ordeal’ by Sand (2009) (costs: increased organisational costs e.g., salary/travel costs to cover 24 hrs; benefits: increased reassurance for patients/carers and less burden/distress; potentially reduced use of ooh GPs/DNs/999-emergency services and/or hospital admissions)

**MECHANISM/S:** where patients/FFC have access to round the clock care **from the specialist palliative care team they have an established relationship with,** they are reassured and can access continuity of care (costs: increased costs of salary/travel costs to cover 24 hrs; specialist staff/training; benefits: increased reassurance for patients/carers and less burden/distress; potentially reduced use of ooh GPs/DNs/999-emergency services and/or hospital admissions; feeling supported/continuity of care)

**OUTCOME/S:** this leads to access to support as/when required and reduces the likelihood of ‘inappropriate’ hospital admissions and/or 999/111 calls (costs: increased organisational costs e.g., salary/travel costs to cover 24 hrs, specialist staff salaries/training; benefits: increased reassurance for patients/carers and less burden/distress; potentially reduced use of ooh GPs/DNs/999-emergency services and/or hospital admissions; feeling supported/continuity of care)

**CMO 24v10hr ‘THE “ORDEAL” OF THE NIGHT – 10hr (DAYS) CARE**

**CONTEXT:** As Dalkin (2018) describes, there is a vulnerability to the night, described as an ‘ordeal’ by Sand (2009) (costs: increased patient/carer burden/distress, feeling unsupported/reassured; potentially reduced use of ooh GPs/DNs/999-emergency services and/or hospital admissions; benefits: reduced organisational costs e.g., salary/travel costs)

**MECHANISM/S:** **where a planned community specialist palliative service exists, with a rapid service for crisis responses overlaid during the day/early evening, strategies can be developed with patients/FFC to predict, plan, and prepare** for overnight, who still have access to DN/GP overnight services (costs: potential workload increase in order to plan (cost to planned service); costs to other services; benefits: patients/carers feel reassured/less ‘burden’, reduced organisational costs e.g., salary/travel costs)

**OUTCOME/S:** this leads to advance management and so avoidance of night-time ‘ordeal’ with no increase in ‘inappropriate’ hospital admissions and/or 999/111 calls (benefits: increased reduced patient/carer burden/distress, feeling supported/reassured; reduced organisational costs e.g., salary/travel costs; reduced use of ooh GPs/DNs/999-emergency services and/or hospital admissions)

**CMO 24v10hr ‘THE “ORDEAL” OF THE NIGHT – RAPID AS OoHs**

**CONTEXT:** Where planned accessible community palliative services are available during the day (costs: reduced organisational costs e.g., salary/travel costs to cover 24 hrs; increased workload need to plan for/avoid the ordeal of the night e.g., cost to planned services)

**MECHANISM/S:** the RRS can operate as a specialist palliative care OoHs service, offering expert care to support services users to avoid/manage the ‘ordeal of the night’ (benefits: allows greater care/response for those in need during ‘the ordeal of the night’ e.g., more directed/appropriate care; in a timely manner) 
**OUTCOME/S:** with positive outcomes for patients/FFC (costs: potential reduction in travel/time spent attending less urgent/appropriate call/outs and/or signposting to other services; benefits: reduced use of ooh/emergency services/hospital admissions; reduced carer/patient burden/distress; feeling supported; timely and specialist response)

**Refined Programme Theory:**

**PT4 12v24hr**

**PT4.1 – 24hr Specialist Support:** Patients approaching and caregivers caring for someone at the end of life, often find the night to be a worrying time due to a lack of service access **(context)**, where patients and informal caregivers have access to 24hr care 7 days a week from the specialist palliative care team who can be contacted through 1 phone number and attend in person if required **(resource),** building relationships, they are reassured and confident to manage dying at home **(response)**, improving perceived well-being **(outcome).** This reduces the likelihood of crisis situations and calls and ‘inappropriate’ hospital admissions **(outcome).**

**PT4.2 – 12hr Planned Specialist Support:** Patients approaching and caregivers caring for someone at the end of life, often find the night to be a worrying time due to a lack of service access **(context)**, where a planned community specialist palliative service exists, with crisis responses during the day (12hr care 7 days a week), strategies can be developed with patients/caregivers to plan and prepare for overnight, with support from OOH DN/GP where necessary, who have shared information systems **(resource).** This builds relationships, and so patients and caregivers feeling reassured and confident to manage dying at home **(response),** improving perceived well-being **(outcome).** This reduces the likelihood of crisis situations and calls and ‘inappropriate’ hospital admissions **(outcome).**

# **IPT7 Timeliness**

Death has become hidden from society and medicalised, but home deaths are the preference of many individuals (context), where there is a timely response guaranteed from RRS (within one hour) (resource), patients and carers feel reassured, supported and confident to provide end of life care (EoLC) at home (reasoning), and this leads to reduced numbers of calls to emergency services and less emergency admissions (outcome).

Rival: Carers perceive the service to be a rapid response line (context) where these expectations are not met (resource) carers lose trust in the service and feel let down (response) which leads to increased carer burden (outcome) and/or increase in emergency calls (outcome).

**Key point:**

What do we mean by ‘timely’ and what do we mean by ‘response’ – together these are critical for reassurance.

**CMO TIMELINESS ‘ONE HOUR RESPONSE’**

**CONTEXT:** The RRS has a direct telephone number available to potential service users (costs: potential increase in self-referrals & needing to prioritise/signpost elsewhere; time spent on ‘inappropriate’ requests; benefits: patients/carers feel reassured and supported and timely response)

**MECHANISM/S**: RRS staff answer the rapid phone number immediately, or, if that is not possible, **attempt to return all calls within one hour**; the speed and directness of this (comparably to other services) is reassuring to service users (in crisis) who then elect to use the RRS for support for EoLC at home (costs: time spent triaging/prioritising calls and signposting; benefits: increased reassurance for patients/carers)

**OUTCOME/S:** service users receive rapid (specialist?) care at home which improves well-being and reduces inappropriate use of other services

**CMO TIMELINESS ‘SERVICE MODELS- CONTACT’**

**CONTEXT**: The RRS has a direct telephone number available to potential service users but differing service models that answer/respond to those calls (1 community specialist palliative care nurse at NRRS; 2x teams of 1 community specialist palliative care nurse and 1 HCA at DRRS) (costs: potential increase in self-referrals & needing to prioritise/signpost elsewhere; time spent on ‘inappropriate’ requests; benefits: for DRRS – patients feel reassured that calls will be dealt with without being ‘triaged’)

**MECHANISM/S:** ***the 2x2 service model of DRRS increases the opportunity for calls into the service to be managed safely and expediently***; the speed and directness of this (comparably to other services) is reassuring to service users (in crisis)  (benefits: patients/carers feel reassured and confident that calls will not be triaged)

**OUTCOME/S**: service users receive rapid specialist care at home which improves well-being and reduces inappropriate use of other services. (benefits: increased well-being of patients and carers through the knowledge that calls will not be triaged/dealt with timely)

**CMO TIMELINESS ‘SERVICE MODELS- RESPONSE’**

**CONTEXT**: The RRS has a direct telephone number available to potential service users but differing service models that answer/respond to those calls (1 community specialist palliative care nurse at NRRS; 2x teams of 1 community specialist palliative care nurse and 1 HCA at DRRS)  (costs: potential increase in self-referrals & needing to prioritise/signpost elsewhere; time spent on ‘inappropriate’ requests; benefits: for DRRS – patients feel reassured that calls will be dealt with without being ‘triaged’)

**MECHANISM/S:** **the service models differ in their responses; with requests for support for personal care and VOED being actioned by DRRS**; the extensive service offered at DRRS provides rapid appropriate care which builds relationships of trust, decreases need for information sharing between teams which could result in lost information, and reduces the potential for carer strain (*potential negative*- time of personal care could strain service if not in last weeks of life) (benefits: increase well-being e.g., reassured that care will be provided as and when needed, less time spent communicating with other services)

**OUTCOME/S**: improves patient/FFC well-being by ‘*alleviat[ing] the multidimensional causes of […] suffering’* (Wee, et al, 2021), halting the escalation of events leading to use of other services, i.e. hospital admission. (*potential negative*- time strain could lead to constraints on other aspects of service) (costs: operating a 2 x 2 service e.g., increased staff/time/travel costs; benefits: benefits: increase well-being e.g., reassured that care will be provided as and when needed, less time spent communicating with other services)

**CMO TIMELINESS ‘AS LONG AS REQUIRED HOME VISIT’**

**CONTEXT:** The RRS has a direct telephone number available to potential service users  (costs: potential increase in self-referrals & needing to prioritise/signpost elsewhere; time spent on ‘inappropriate’ requests; ‘triaging’ calls)

**MECHANISM/S:** RRS staff, who are specialists in EoLC, with expertise in supporting people to die at home, will answer the direct line within one hour and **respond with a visit to a variety of difficult/crisis situations (symptom management, distress, discomfort)** at the service users home and **stay for as long as situation** takes to manage with no time deadline (i.e. not a 15/30min timed stay) (benefits: increased reassurance/support/well-being that calls will be dealt with timely and multiple aspects of care provided in ‘one call’ reducing distress/anxiety)

**OUTCOME/S**: service users are supported quickly and expertly to manage with positive outcomes for patient and FFC with an associated reduction in the use of other inappropriate services.  (costs: increased time spent per visit/per patient; potentially decreasing time/calls to other patients benefits: increased reassurance/support/well-being that calls will be dealt with timely and multiple aspects of care provided in ‘one call’ reducing distress/anxiety)

**CMO TIMELINESS ‘PHONE A SPECIALIST’**

**CONTEXT:** Recent generations have less experience of death and dying so are uncertain, fearful, and unsure when overseeing and supporting process at home

**MECHANISM/S**: The RRS has a direct telephone number available to all potential service users, **RRS staff, who are specialists in EoLC, with expertise in supporting people to die at home, are available to answer this rapid phone line to any questions from concerned (and often stressed) service users**, armed with that knowledge, service users can take appropriate action and/or are reassured, avoiding the need for home visits or escalation of situations  (costs: potential increase in self-referrals & needing to prioritise/signpost elsewhere; time spent on ‘inappropriate’ requests; ‘triaging’ calls; benefits: increased well-being e.g., reducing stress/distress and increasing feelings of reassurance and support from ‘expert staff)

**OUTCOME/S:** all service users well-being is improved (benefits: increased well-being e.g., reducing stress/distress and increasing feelings of reassurance and support from ‘expert staff)

**CMO TIMELINESS ‘CHECKING-IN CALLS’**

**CONTEXT:** Where a vulnerable situation has been detected by RRS staff, i.e. rapid deterioration, carer concern/strain) (costs: time spent ascertaining deterioration)

**MECHANISM/S:** RRS staff ensure that the patient/FFC are directly contacted, ‘checking-in’ with them, avoiding a situation escalating as the patient/FFC have felt unable/unsure to call in (time spent ‘checking in’ and ascertaining deterioration; benefits: impact on well-being e.g., reducing stress/distress and increasing support/reassurance)

**OUTCOME/S**: crisis are potentially averted and timely advice and support accessed by service users with positive impact on their well-being.  (benefits: impact on well-being e.g., reducing stress/distress and increasing support/reassurance)

**Refined Programme Theory:**

**PT3 Timely Response**

Death has become hidden from society and medicalised, but home deaths are the preference of many individuals; this means patients and caregivers may find dying at home emotionally daunting and practically challenging **(context).** Where there is a timely response guaranteed from a specialist palliative care service (one hour) with a home visit with no time deadline (i.e., not a 15/30 min timed stay) if required, and/or a telephone response for support for difficult or crisis situations (symptom management, distress, discomfort) **(resource),** provides emotional and practical reassurance, leading to improved confidence to patients and caregivers **(response).** This leads to less (attempts) at use of other generalist or emergency services **(outcome)** and/or improves perceived wellbeing for patients and caregivers **(outcome).**

# **IPT8 Who (at home/from service)**

The provision of EoLC at home requires internal and external support (context), when the internal supporter(s) (FFC) is confident and committed to EoLC at home (as perceived by themselves and the patient) (resource-reasoning) and trusts in the skills, supplies, service, personhood, of the external support (RRS) (resource-reasoning), the RRS will become the service of choice, avoiding use of the emergency services or DN's (outcome-1) and/or supporting death at home (outcome-2)

**Key point: Who is at home** (caregiver + other family/dependants?) and what resources/needs do they have? +/ **Who is coming in from the service** (staff competencies/capabilities/cultural)

**CMO WHO- AT HOME ‘FEAR OF BURDEN’**

**CONTEXT/S:** Different families have difference needs, dynamics, and capacity to care (costs: expert knowledge/skills to identify/capacity to care for different needs)
**MECHANISM/S**: whether their supporters/caregivers agree or not, patients fear they will be a burden on those who would care for them at home, (**negative**- and so do not engage with the service and would prefer to go into a hospice/hospital) (**positive**- this fear is quelled by expertise and time availability of RRS staff) (costs: where non-engagement, increased costs to other/emergency services: benefits: increased well-being for users e.g. reassurance/support, reducing distress/fear)

**OUTCOME/S:** lack of engagement with service and use of other services OR positive engagement with RRS

**CMO WHO- AT HOME ‘FEAR AND CARE’**

**CONTEXT/S:** With low death literacy in the UK, managing death and dying at home can be a frightening, difficult experience, perhaps particularly when familial roles are changing (i.e., child/sibling/spouse-caregiver) (costs: expert knowledge/skills to identify/capacity to care for different needs)
**MECHANISM/S:** accessible, available, expert support and care from RRS staff builds knowledge, skills and experience of supporters and caregivers at home; and/or provides caregivers with direct support to maintain their capacity to care; consequently, caregivers are then confident and able to adapt and support end of life care at home (benefits: increased well-being through feeling supported/reassured/confident to manage care at home)

**OUTCOME/S:** the patient can realise their wish to die at home with a reduction in hospital admissions at the end of life (benefits: increased well-being through feeling supported/reassured/confident to manage care at home; preferred place of death met e.g. at home)

**CMO WHO- AT HOME ‘CHANGING MINDS’**

**CONTEXT/S**: Supporting a patient to die at home requires multiple resources, those which can be provided by community end of life services, and those which require supporters (be they friends, family, carers) (costs: staffing, training, travel; carer costs e.g., time off work, childcare, out of pocket expenses, friends)

**MECHANISM/S**: patients and/or supporters **may not be available or able to manage** and they may change their minds and want to change the place of death from home to a hospice or elsewhere, ***the specialist expertise of RRS staff allows them to ascertain whether this is in response to a challenge they can support to overcome or whether the place of death does require changing***, and provide assistance to access alternate care if that is appropriate (costs: staffing, training/expertise, time resources)

**OUTCOME/S**: positive well-being outcomes for patient and caregivers (benefits: carer/patient well-being, being able to die where they choose, feeling supported)

**CMO WHO- AT HOME ‘MINIMUM SUPPORT’**

**CONTEXT:** Supporting someone to die at home where that is their wish requires a minimum level of support – from outside agencies coming into the home, and supporters within the home (costs: staffing, training, travel; carer costs e.g., time off work, childcare, out of pocket expenses, friends)

**MECHANISM/S:** the RRS is available for rapid/crisis calls to support patients and caregivers at home (i.e., they cannot provide caseload, planned, permanent care), ‘other’ care is provided by DNs, GPs, Social Care, caregivers; ***without enough ‘other’ care (which is dependent on available social networks of support, which may be especially difficult with a rapid deterioration, the RRS cannot be used*** (costs: ‘other’ costs to services e.g. OoH gps, DNs etc, carers/friends time, well-being)

**OUTCOME/S**: with negative potential well-being outcomes for patient/caregiver, and dis-engagement from the service (costs: negative impact on patient/carer well-being, distress/stress/fear/anxiety, feeling unsupported; greater reliance on friends/other support)

**CMO WHO- FROM SERVICE ‘RRS STAFF COMPETENCIES’**

**CONTEXT/S**: The RRS staff have multiple key competencies (***inc. expertise in PEoLC, prescriber status, cultural awareness, initiative, and problem-solving skills, teaching, supporting and empowering carers, ability to act as conduit***) (costs: specialist skills expertise, staffing e.g.., grade 7; training)

**MECHANISM/S**: and so, can provide specialist advice, support and guidance which provides reassurance, reduces distress, increases trust, and provides expert care provision to service users

**OUTCOME/S**: engagement with service (over alternatives) with positive health and wellbeing outcomes for service users (benefits: positive impact on patient/carer well-being, feeling supported, trust and building relationships, positive interactions with RRS; reduced burden on carers e.g., financial and emotional)

**Refined Programme Theory:**

**PT6 Responsive Skilled Support**

Supporting death and dying at home, necessitates informal caregivers to have access to several resources (economic, physical, social, cultural, and emotional) which differ in each individual circumstance **(context)**. Where specialist end of life care services, with prescribing capabilities, cultural competences and inclusive practices, are available to respond quickly and flexibly, without time limits, to all these potential support needs **(resource)**, caregivers and services can ‘work together’ **(resource),** and patients and caregivers feel reassured, confident, and capable to continue to provide care at home, or to change their minds/course and access in-patient or Hospice care if appropriate **(response).** This leads to improved perceived well-being of patients and caregivers **(outcome)** and improved opportunities for death at home **(outcome)**
